# St. Mary's Paddington

**Published:** 1902-03-22

**Authors:** 


### ST. MARY'S PADDINGTON.

### RAPID PROCEEDINGS.

The Governors of St. Mary's Hospital, Paddington, are not likely to be visited by an overwhelming contingent of the Press. One such representative, arriving seven minutes late on Friday, 14th inst., was greeted with the information that "it was all over and done." And the Board of management was serenely drinking its tea.

"We used to work up our meetings," said the secretary, who had returned to his office, "but we have given it up, It didn't pay. We used to go to a great deal of trouble to get a good platform, and even then only half a dozen came. So now we don't trouble. Annual meetings are the purest formality, and we consider them practically a farce. Why should people come here and pass a report which they have read already, and move resolutions on matters they know all about? We know they do read the report from the splendid support they give us, but we make no effort to get them here. We simply advertise the meeting, and the Board of Management does the business. The chairman moved the adoption of the report and the deputy chairman seconded. Ten were present this afternoon."

